# A Benign Mimic of Dangerous Neck Pathology: A Case Report of Longus Colli Calcific Tendonitis

**DOI:** 10.5811/cpcem.2022.6.56243

**Published:** 2022-12-29

**Authors:** Alyse Volino, Stephanie Smith

**Affiliations:** Cooper University Hospital, Department of Emergency Medicine, Camden, New Jersey

**Keywords:** case report, neck pain, tendonitis

## Abstract

**Introduction:**

Longus colli calcific tendonitis (LCCT) is a calcium deposition disease that causes acute or subacute atraumatic neck pain. It is important for the emergency physician to consider LCCT in the differential diagnosis because the clinical presentation of this benign condition may mimic life-threatening disease processes that require invasive diagnostic measures.

**Case Report:**

We present a case of a 63-year-old female with atraumatic right-sided neck pain. On exam she had tenderness to palpation in the neck, as well as difficulty ranging her neck and opening her mouth. She underwent computed tomography of her neck with intravenous contrast, which showed calcific tendonitis of the longus colli muscle with retropharyngeal edema. She was seen by otolaryngology, underwent nasopharyngolaryngoscopy, and ultimately was discharged with antibiotics and corticosteroids.

**Conclusion:**

The presentation of LCCT can mimic symptoms of dangerous causes of neck pain including retropharyngeal abscess and meningitis. Early diagnosis in the ED can potentially avoid more invasive diagnostic and therapeutic measures. While LCCT is thought to be self-limiting, it can be treated with non-steroidal anti-inflammatory medications and corticosteroids. If pain is controlled, patients can be discharged from the ED with no specialist follow-up required.

## INTRODUCTION

Acute calcific tendonitis of the longus colli muscle (also called retropharyngeal calcific tendonitis) is a benign and self-limiting condition that presents similarly to potentially dangerous disease processes. Patients with longus colli calcific tendonitis (LCCT) often present with neck pain, neck stiffness, and odynophagia, and at times will also have headache and subjective fevers.^1^–^3^ This constellation of symptoms could be consistent with several potentially devastating diagnoses that the emergency physician must consider, including retropharyngeal abscess, mastoiditis, and meningitis. It is, therefore, important to recognize LCCT as a possible diagnosis in the patient with atraumatic neck pain and stiffness, as timely diagnosis with this benign condition may avoid more aggressive testing and treatment.

## CASE REPO/RT

A 63-year-old female presented to the emergency department (ED) with a chief complaint of right-sided neck pain. She had a past medical history of non-insulin-dependent diabetes, hypertension, and hyperlipidemia, which were controlled with medications. The neck pain had been worsening for the prior five days and was located behind her right ear with radiation to the anterior and posterior soft tissue of the right side of the neck. She had been seen in the ED with neck pain the day before and was discharged, but she returned with progressive symptoms that now included sore throat, pain with swallowing, and difficulty moving her neck. She denied history of trauma, fevers, nasal congestion, headache, recent dental work or tooth pain, and ear pain or discharge.

On physical exam, she was afebrile with normal vital signs. She was in no acute distress and had no trouble managing her secretions. She had some difficulty opening her mouth fully due to pain. Active and passive range of motion of the neck was limited due to pain. She had tenderness to palpation on the right side of her neck, both anterior and posterior to her ear. There was no tenderness to palpation in the midline over the cervical spine. She had no asymmetry in the neck or palpable lymph nodes and no overlying skin changes. Her tympanic membranes were normal bilaterally and she had no swelling, pain, erythema, or drainage in her pinna or external auditory canal.

**Image 1:**
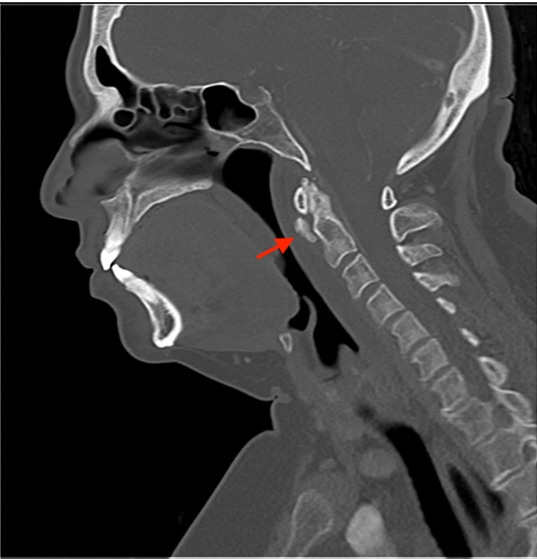
Sagittal computed tomography image in bone window showing focal calcification (arrow) in the longus colli tendon at the level of the first and second cervical vertebrae.

**Image 2:**
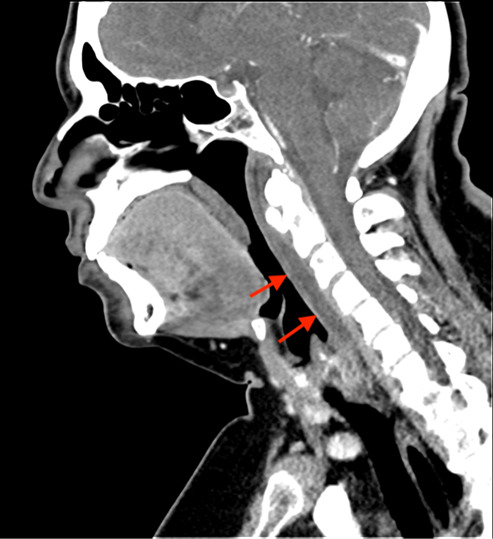
Sagittal computed tomography image in soft tissue window showing diffuse retropharyngeal edema (arrows).

Laboratory results showed white blood cell count (WBC) of 11.59 × 10^9^ cells per liter (x 10^9^/L) (reference range: 4.50 – 11.0 × 10^9^/L). Erythrocyte sedimentation rate and C-reactive protein were not sent. Computed tomography (CT) of the neck with intravenous (IV) contrast was done, as the differential diagnosis included mastoiditis, retropharyngeal abscess, and other deep space neck infection. Computed tomography results showed prominent amorphous calcification inferior to the anterior arch of the first cervical vertebrae, highly suggestive of calcific tendonitis of the longus colli muscle (Image 1), with diffuse retropharyngeal edema (Image 2).

*CPC-EM Capsule*

What do we already know about this clinical entity?

*Longus colli calcific tendonitis (LCCT) is caused by calcium crystal deposition in the longus colli muscle, which can cause neck pain and neck stiffness.*

What makes this presentation of disease reportable?

*The presentation of LCCT, including atraumatic neck pain, neck stiffness, and subjective fevers, mimics pathologies like retropharyngeal abscess or meningitis.*

What is the major learning point?

*LCCT causes acute to subacute neck pain that mimics life-threatening pathology, but is a benign, usually self-limiting, condition.*

How might this improve emergency medicine practice?

*Diagnosing LCCT in the emergency department can potentially avoid more invasive diagnostic testing, therapeutic interventions, and even hospital admission.*

The patient was monitored in the observation unit and was seen by the otolaryngology team, which performed nasopharyngolaryngoscopy the next day to assess for airway obstruction in the setting of retropharyngeal swelling. At this time, her WBC count had increased to 14 × 10^9^/L. Nasopharyngolaryngoscopy showed mild bulging of the left pharyngeal wall with no obstruction of the glottic airway and no concern for abscess or fluid collection. The edema was thought to be reactive secondary to tendonitis rather than due to infectious etiology. However, because of the elevation in WBCs in the setting of retropharyngeal edema, the otolaryngology team recommended empiric treatment with antibiotics. The patient was discharged with a 10-day course of amoxicillin/clavulanate and a 10-day course of prednisone.

## DISCUSSION

Longus colli calcific tendonitis occurs due to calcium hydroxyapatite crystal deposition within the longus colli muscle fibers and tendon. This type of calcium deposition disease is more commonly found in the shoulder joint and has been less well-described in the longus colli muscle.^4^–^6^ Symptoms of pain and stiffness are thought to be due to a foreign body inflammatory response to the presence of calcium crystals.^2^ The exact etiology of the calcium crystal deposition is unknown. Hypotheses have included repetitive trauma, ischemia, and tendon degeneration, although most patients present with no direct preceding incident to explain symptoms.^2^,^3^,^6^,^7^

The longus colli muscle is located along the ventral aspect of the cervical vertebrae in the prevertebral space, extending from the first cervical vertebra to the third thoracic vertebra. The muscle functions in neck flexion and rotation. It is made up of three parts: vertical; inferior oblique; and superior oblique, with the superior aspect most commonly affected by calcific deposits.^2^,^3^,^8^,^9^ Although the muscle and, therefore, the calcific deposits are located in the prevertebral space, there is often associated edema (and even at times fluid collection) in the adjacent retropharyngeal space, as was seen in our patient. Affected patients are usually in the third through sixth decades of life, but have been described from ages 21–81.^1^–^3^,^6^–^10^

The typical presentation of LCCT includes acute or subacute nontraumatic neck pain, neck stiffness, and odynophagia. Patients also have been described reporting headaches and subjective fevers. The WBC count is often normal to slightly elevated, and erythrocyte sedimentation rate may be elevated as well.^1^–^3^ The clinical picture often prompts a work-up for disc herniation or for infectious etiology of symptoms, including retropharyngeal abscess, mastoiditis, and meningitis.^1^,^2^,^9^–^11^

Computed tomography is the optimal imaging choice for diagnosing LCCT. It will identify the calcific deposits and will characterize associated retropharyngeal edema, also allowing for distinction between this diagnosis and retropharyngeal abscess if IV contrast is used. Radiograph of the cervical spine does not always reveal the calcific deposits and may result in a missed diagnosis if used in isolation. Magnetic resonance imaging will identify the soft tissue edema or fluid collection but will not clearly elucidate the calcific deposits, which could also result in a missed diagnosis.^3^,^7^

The characteristic CT findings include calcific deposits in the prevertebral space, ventral to the first and second cervical vertebrae, localized to the longus colli muscle. Computed tomography often will also show surrounding edema in the prevertebral or retropharyngeal space.^1^–^3^,^7^,^9^–^12^ Retropharyngeal effusion without surrounding structural enhancement has also been described, with the lack of an enhancing wall differentiating this fluid collection from abscess.^7^,^9^

Once the diagnosis of acute calcific tendonitis of the longus colli muscle has been made, conservative management is appropriate, with resolution of symptoms usually occurring in 1–2 weeks. The condition appears to be self-limiting, in that no serious complications or progressions of the disease have been reported, although it is widely acknowledged that this disease process is likely under-diagnosed and under-reported. Non-steroidal anti-inflammatory drugs and corticosteroids are frequently used to help improve symptoms more rapidly.^1^,^2^,^4^,^7^,^9^–^12^ Treatment with antibiotics is unnecessary. If the diagnosis is made in the ED, patients can likely be discharged if pain is controlled. No specific specialist follow-up is required.

## CONCLUSION

Neck pain is a common chief complaint in the ED that can cause debilitating pain for patients. We describe a case of neck pain caused by acute calcific tendonitis of the longus colli muscle, which was diagnosed in the ED. This condition is benign and self-limiting, but its presentation can mimic that of more dangerous head and neck pathology. It is, therefore, important for the emergency physician to consider this diagnosis when evaluating atraumatic neck pain and stiffness. Prompt diagnosis with this tendonitis can avoid more aggressive testing and invasive treatment modalities, as patients can often be discharged with oral medications to treat their pain. By avoiding further testing and hospital admission, healthcare costs are reduced.
